# Indoleamine 2,3-dioxygenase (IDO) is frequently expressed in stromal cells of Hodgkin lymphoma and is associated with adverse clinical features: a retrospective cohort study

**DOI:** 10.1186/1471-2407-14-335

**Published:** 2014-05-15

**Authors:** Ji-Young Choe, Ji Yun Yun, Yoon Kyoung Jeon, Se Hoon Kim, Gyeongsin Park, Joo Ryoung Huh, Sohee Oh, Ji Eun Kim

**Affiliations:** 1 Department of Pathology, Seoul National University Bundang Hospital, Seongnam-si, Korea; 2 Department of Pathology, Seoul National University Hospital, Seoul, Korea; 3 Department of Pathology, Yonsei University College of Medicine, Seoul, Korea; 4 Department of Pathology, Seoul St. Mary’s Hospital, The Catholic University of Korea, Seoul, Korea; 5 Department of Pathology, Asan Medical Center, Seoul, Korea; 6 Department of Biostatistics, Seoul National University Boramae Hospital, Seoul, Korea; 7 Department of Pathology, Seoul National University Boramae Hospital, Seoul, Korea

**Keywords:** Hodgkin disease, Indoleamine-pyrrole 2,3-dioxygenase, Macrophages, Stromal cells, Tumor microenvironment, Epstein-barr virus infections, Pathology

## Abstract

**Background:**

Regulation of tumor microenvironment is closely involved in the prognosis of Hodgkin lymphoma (HL). Indoleamine 2,3-dioxygenase (IDO) is an enzyme acting as immune modulator through suppression of T-cell immunity. This study aims to investigate role of IDO in the microenvironment of HL.

**Methods:**

A total of 121 cases of HL were enrolled to do immunohistochemistry for IDO, CD163, CD68, CD4, CD8, and FoxP3. Positivity was evaluated from area fractions or numbers of positive cells using automated image analyzer. Correlations between IDO expression and various cellular infiltrates and clinicopathologic parameters were examined and survival analyses were performed.

**Results:**

IDO was expressed in histiocytes, dendritic cells and some endothelial cells with variable degrees, but not in tumor cells. IDO positive cells were more frequently found in mixed cellularity type than other histologic types, and in cases with EBV+, high Ann Arbor stages, B symptoms, and high IPS (all p < 0.05). High IDO expression was associated with inferior survival (p < 0.001) and reflects an independent prognostic factor in nodular sclerosis HL.

**Conclusions:**

This is the first study suggesting that IDO is the principle immunomodulator and is involved to adverse clinical outcomes of HL.

## Background

The pathology of Hodgkin lymphoma (HL) is characterized by the relative paucity of tumor cells and an abundance of reactive background cells. The composition and frequencies of the reactive cellular milieu of HL varies considerably among individual patients and histologic types and at different stages throughout the course of the disease [1]. As there has recently been a renewed interest in the role of the tumor microenvironment, the reactive cells in HL are now thought to be active participants in tumor progression and immune escape [2]. The cellular micromilieu of HL can be divided into two groups: inflammatory cells and stromal cells. Among these cells, macrophages and T cells, particularly regulatory T cells (Treg), have been consistently scrutinized in regard to patient outcomes [2]. Although many studies regarding the microenvironment of HL have been conducted over the years, most of the regulatory mechanisms of Hodgkin Reed Sternberg (HRS) cells on the surrounding tumor microenvironment remain elusive due to the complex interactions that occur among several soluble and cellular factors.

Indoleamine 2,3-dioxygenase (IDO) is a tryptophan catabolic enzyme that degrades tryptophan via the kynurenine pathway [3]. This enzyme is involved in various pathophysiological processes such as infection, autoimmunity and anti-tumor defense [4]. It is a potent immune system modulator produced by macrophages and dendritic cells, and it suppresses T cell immunity through the inhibition of effector T cell function and the induction of CD4 + CD25 + FOXP3+ Tregs [5,6].

Until now, most studies regarding IDO were conducted in an immunologic context such as the fields of transplantation or autoimmunity, and little effort has been expended on the role of IDO in the tumor microenvironment. Several studies have reported that IDO expression correlates with poor clinical outcome in some cancer types including colorectal, endometrial, ovary and lung cancers and malignant melanoma [7-10]. With regard to hematological malignancies, very few attempts have been made to elucidate the effects of IDO on the tumor microenvironment. Except for a few studies of diffuse large B-cell lymphomas, the tumor microenvironment of HL has not yet been explored despite the importance of non-tumor components in clinical outcome [11].

Here, we aimed to evaluate the role of IDO in the microenvironment of HL. We have identified IDO positive cells in HL tissue and analyzed its effects on the infiltration of other inflammatory cells, patients’ clinicopathologic features and survival.

## Methods

### Patients

A total of 121 consecutive HL patients with available tissue were enrolled in this study from the Boramae Medical Center and the Seoul National University Hospital. Tissues of these patients were collected from stored paraffin blocks which were originally obtained at the time of initial diagnosis of HL. Histologic features and Epstein-Barr virus (EBV) status were reviewed by two pathologists based on the current WHO criteria. Clinical data including age, sex, Ann Arbor stages, B symptoms, bulky disease, Human immunodeficiency virus (HIV) status, International prognostic score (IPS), lactate dehydrogenase (LDH), initial blood lymphocyte and monocyte counts, treatment response, and survival data were obtained from electronic medical records. This study was granted by the Institutional Review Board of the Seoul National University Boramae Hospital.

### Tissue microarray construction

Tissue microarray (TMA) blocks were manufactured for immunohistochemistry (IHC). Two core tissues containing the most representative tumor areas (3 mm or 5 mm in diameter) were taken from the individual donor blocks and arranged into new recipient TMA paraffin blocks using a trephine apparatus.

### Immunohistochemistry (IHC)

Immunohistochemical staining for IDO (Millipore, Billerica, MA, USA), CD68 (Dako, Carpinteria, California, USA), CD163 (Novocastra, Newcastle, UK), CD4 (Novocastra), CD8 (Dako), and FOXP3 (Abcam, Cambridge, UK) was performed on the TMA blocks following a standard protocol using a Ventana Automated Immunostainer (Ventana, Benchmark, Tuscan, AZ USA). After deparaffinization, heat-induced antigen retrieval was performed using citrate buffer, pH 6.0 (CC1 protocol, Ventana). Reactivity was detected using the Ultra-View detection kit (Ventana).

### Double IHC

To identify the lineage of IDO producing cells, double IHC staining against IDO and CD68/CD163 was carried out in the most representative cases of mixed cellularity (MC) and nodular sclerosis (NS) subtypes. A peroxidase system with 3,30-diaminobenzidine and hydrogen peroxide was applied for detection of the first primary antibody, and an alkaline phosphatase system (Bond Polymer Refine Red Detection, Leica, Wetzlar, Germany), for the second primary antibody.

### Automated image analysis

Semi-quantitative interpretation of IHC was performed using automatic image analysis. Image J software (NIH Image, Bethesda, MD, USA) was used to calculate the area and number of positive cells with cytoplasmic (IDO, CD68, CD163) and nuclear staining (FOXP3) patterns. Aperio Image Analyzer software (Aperio, Vista, CA, USA) was used to count cells with membranous staining (CD4, CD8). The area of the frame used for the counts was approximately 1 mm^2^ (996,944 μm^2^), and multiple fields were sampled in the areas which exhibited the richest HRS cell abundance. To standardize tumor area and enable valid comparisons, all cores were manually reviewed at the same time. The fraction of the total area containing positive cells (a total area of 1 mm^2^) was calculated for IDO, CD68, or CD163, and the number of positive cells was determined for CD4, CD8, and FOXP3 staining.

### Statistical analysis

Statistical analyses were performed using the Statistical Package for Social Sciences software, version 20.0, for Windows (IBM, IL, USA), and *p* values less than 0.05 were considered statistically significant based on a two-sided statistical analysis. Non-parametric correlation between IDO expression and various cellular infiltrates was tested via Spearman’s rho, and Mann–Whitney or Kruskal-Wallis tests were used to compare groups with different clinicopathologic variables. For survival analyses, patients were divided into two groups according to the expression of IDO and other cellular infiltrates. Cutoff values were chosen either by maximum value of Youden’s index (J = sensitivity + specificity-1) from the receiver-operating characteristics (ROC) curves or by median values when ROC curves were not available. Survival time was defined to be the period of time in months from the date of diagnosis to the date of death from any cause. Progression time was defined as the period of time in months from the date of diagnosis to the date at which progression of disease was clinically identified by computed tomography or positron emission tomography. Overall survival (OS) and progression-free-survival (PFS) were compared using the Kaplan-Meier method with a log-rank test. A multivariate Cox proportional hazards model with a backward elimination was performed.

## Results

### Patient characteristics

Patients’ overall clinicopathologic characteristics and the results of IHC are summarized in Table 1. The male to female ratio was 2:1, and the age distribution was 10–80 years (mean = 38.2 years). Among 121 HL cases, there were 5 nodular lymphocyte predominant (NLP), 64 NS, 46 MC, 1 lymphocyte-depleted, 1 lymphocyte-rich, and 4 unclassifiable cases. Forty-nine patients (42.6%) had high Ann Arbor stages (defined as stage III or IV), and approximately one third (31.8%) of all patients experienced B symptoms. One third (29.4%) had high IPS (≥3). EBV was detected in 43 of 96 cases (44.8%), and HIV infection was observed in 3 patients. Most patients received chemotherapy with standard ABVD regimen (adriamycin, bleomycin, vinblastine, and dacarbazine) with/without adjuvant radiotherapy. A few patients received chemotherapy with MOPP regimen (nitrogen mustard, vincristine, procarbazine, and prednisone) or radiotherapy alone. Twenty-five of the 114 patients (22.2%) died within the 7-year median follow-up period (range, 0.3 to 15.7 years). Twenty-one patients (21.4%) experienced tumor relapse, and twelve (12.2%) had progressive disease.

**Table 1:** Demographics and the distribution of IDO expression

**Variable**	**Total N (%)**	**IDO expression**	
		**Mean**	**P-value**
**Age, years**	121		
<50	85 (70.2)	7.0	<0.001
≥50	36 (29.8)	20.2	
**Sex**	121		
Female	41 (33.9)	7.4	<0.001
Male	80 (66.1)	12.8	
**Histologic subtype**	121		
NLP	5 (4.1)	3.3	<0.001
NS	64 (52.9)	5.8	
MC	46 (38.0)	19.2	
LD	1 (0.8)	15.6	
LR	1 (0.8)	1.1	
Unclassifiable	4 (3.3)	8.9	
**Ann Arbor stage**	115		
I-II	66 (57.4)	6.9	0.004
III-IV	49 (42.6)	17.3	
**B symptoms**	107		
Yes	34 (31.8)	16.2	0.454
No	73 (68.2)	9.1	
**Bulky disease**	104		
Yes	8 (7.7)	11.8	0.826
No	96 (92.3)	11.8	
**IPS**	116		
≤2	82 (70.7)	7.5	0.013
>2	34 (29.3)	20.6	
**EBV status**	96		
Positive	43 (44.8)	17.4	0.069
Negative	53 (55.2)	8.7	
**HIV status**	121		
Positive	3 (2.5)	58.6	0.001
Negative	118 (97.5)	9.7	
**Treatment response**	98		
Success	65 (66.3)	10.0	0.098
Relapse	21 (21.4)	11.0	
Progression	12 (12.2)	26.4	

IDO, indoleamine 2,3-dioxygenase; NLP, nodular lymphocyte predominant; NS, nodular sclerosis; MC, mixed cellularity; LR, lymphocyte-rich; LD, lymphocyte-depleted; IPS, international prognostic score.

### IDO expression via IHC

**Figure 1:**
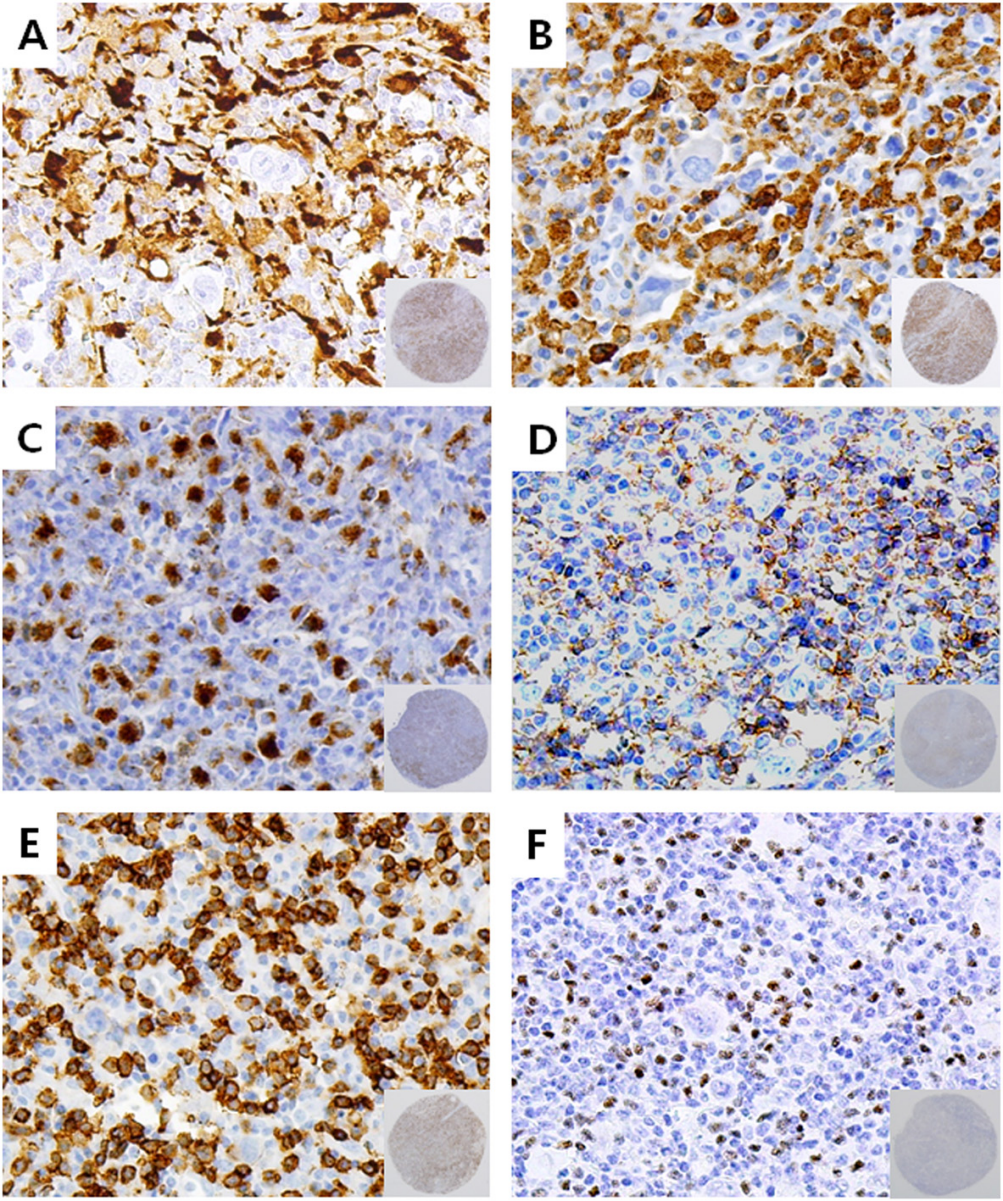
**Representative features of immunohistochemistry in Hodgkin lymphoma (HL).** (Inlet: an entire core tissue of tissue microarray) IDO (indoleamine 2,3-dioxygenase) is highly expressed in macrophages, dendritic cells, and some endothelial cells, but not in Hodgkin Reed Sternberg cells (HRS) or lymphocytes **(A)**. CD68+ macrophages are found near HRS **(B)**. CD163 is positive in both macrophages and some dendritic cells **(C)**. CD4+ **(D)**, CD8+ **(E)**, or FOXP3+ **(F)** T-cells are found in the background of HL.

**Figure 2:**
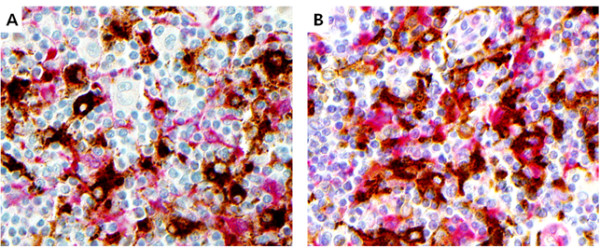
**Double immunohistochemistry with IDO and CD68/CD163 in classical Hodgkin lymphoma.** Positive immunoreactivity of IDO (red color) is colocalized with CD68 **(A)** or CD163 **(B)** (both, brown color) in the cytoplasm of macrophages or dendritic cells.

IDO expression was observed in macrophages, dendritic cells and vascular endothelial cells, but not in HRS cells or lymphocytes (Figure 1). In double IHC assay, co-localization of IDO and CD68, or IDO and CD163 was demonstrated (Figure 2). The staining pattern of IDO was largely cytoplasmic with focal nuclear staining in some dendritic cells. The fraction of the area containing IDO positive cells was variable and ranged from less than 0.1% to 83.4% (median, 1.9%).

### IDO expression and clinicopathologic variables

Associations between IDO expression and clinicopathologic variables including bulky disease and B symptoms are listed in Table 1. Patients with high IDO expression correlated with advanced Ann Arbor stages (p = 0.004), high IPS (p = 0.013), old age (p < 0.001) and male sex (p < 0.001). Regarding histologic types, nodular lymphocyte predominant HL displayed the lowest IDO expression (mean area fraction = 3.3%), and the mixed cellularity type exhibited the highest expression of IDO (mean = 19.2%). The nodular sclerosis type also showed very low levels of IDO expression (mean = 5.8%) (p < 0.001).

EBV positive HL showed a tendency towards high IDO expression (p = 0.069), and all three HIV-positive patients showed extremely high IDO expression (mean = 58.6%) (p = 0.001). In HIV-negative classical HL, EBV positive cases showed significantly higher expression of IDO than EBV-negative cases (p = 0.044, Additional file 1: Table S1).

### IDO expression and cellular infiltrates

The results of the correlations between IDO expression and various cellular infiltrates in classical HL (cHL) with immunocompetent patients (excluding NLPHL and HIV patients) are summarized in Table 2. Infiltration of CD163+ and CD68+ cells correlated with IDO expression (both p < 0.001). Upon histologic examination, CD163 was positive primarily in macrophages but also was present in some dendritic cells; however, CD68+ cells were not shown the dendritic feature in IHC staining. Therefore, the fraction of the area containing CD163 positive cells (median, 9.1%; range, 0.1%-92.2%) was higher than the fraction of the area containing CD68 positive cells (median, 1.3%; range, 0.1%-28.2%). In addition, peripheral blood monocyte counts at the time of initial diagnosis also correlated with IDO (p = 0.015), CD163 (p = 0.035), and CD68 (p = 0.011) expression.

**Table 2:** Correlation between IDO expression, cellular infiltration and EBV positivity in HIV negative classical Hodgkin lymphoma

		**CD163**	**CD68**	**Mono**	**CD8**	**CD4**	**FOXP3 /CD4**	**Subtype**	**EBV**
**IDO**	r	0.439	0.493	0.225	0.368	-0.200	0.210	0.433	0.158
	P-value	<0.001	<0.001	0.026	<0.001	0.060	0.048	<0.001	0.150
**CD163**	r		0.668	0.179	0.194	0.102	-0.116	0.234	0.092
	P-value		<0.001	0.107	0.084	0.360	0.301	0.028	0.434
**CD68**	r			0.229	0.240	0.206	-0.084	0.197	0.118
	P-value			0.044	0.034	0.069	0.461	0.072	0.328
**Monocyte**	r				0.109	-0.183	0.211	0.190	0.170
	P-value				0.329	0.094	0.053	0.062	0.129
**CD8**	r					0.123	0.009	0.368	-0.103
	P-value					0.264	0.936	<0.001	0.397
**CD4**	r						-0.788	-0.261	-0.197
	P-value						<0.001	0.014	0.092
**FOXP3** **/CD4**	r							0.090	0.164
	P-value							0.402	0.164
**Subtype**	r								0.274
	P-value								0.011

r, correlation coefficient; IDO, indoleamine 2,3-dioxygenase; EBV, Epstein-Barr virus; HIV, human immunodeficiency virus.

Expression of IDO was positively correlated with the number of CD8+ T cells (p < 0.001) and negatively correlated with the number of CD4+ T cells (p = 0.020). Infiltration of FOXP3+ Treg cells did not correlate with IDO expression (p = 0.795). However, the percentage of Treg within CD4+ T cells (FoxP3/CD4+ ratio) correlated with IDO in patients with limited disease status (stage I, II) (p = 0.002).

### Survival analysis

For univariate analyses, the patients were divided into two groups according to the cutoff values of various cellular markers. ROC curves were generated, areas under the curve (AUCs) were measured for IDO, CD163, and CD68, and the cutoff points were determined as 26%, 33%, and 5%, respectively. For the CD4, CD8, FoxP3/CD4, and peripheral blood monocyte counts, median values were chosen as the cutoff values because we were unable to evaluate ROC curves and AUCs for these parameters. We analyzed the patients’ survival among the cohort of HIV-negative classical HL (cHL), and also separately analyzed among NS and MC subtypes.

**Figure 3:**
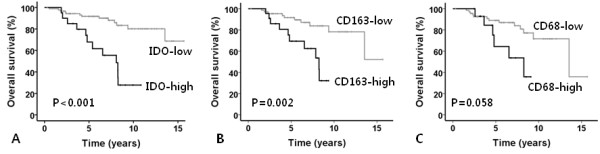
**Survival plots of classical Hodgkin lymphoma tested by Kaplan-Meier method according to IDO expression.** High IDO expression was associated with poor overall survival in HIV-negative classical Hodgkin lymphoma patients. **(A)**. In addition, Infiltration of macrophages or dendritic cells represented by CD163 **(B)** or CD63 **(C)** was associated with adverse outcome.

The results of the univariate survival analyses are summarized in Table 3. Patients with high IDO expression, frequent infiltration of CD163+ or CD68+ cells showed significantly shortened OS (p < 0.001, p = 0.002, p = 0.058, respectively) (Figure 3). The 5-year OS rate was much lower for patients with high IDO positivity (67.8%) than for those with low IDO positivity (91.7%). In a subgroup analysis based on histologic types, high IDO expression was associated with poor overall survival in both MC and NS subtypes (both p = 0.017). In the multivariate analyses among HIV- negative cHL cases, the effect of IDO on patients’ OS was not significant, although still trended toward negative prognostic indicator (p = 0.111). However, in NS subgroup, IDO was still an independent prognostic factor (p = 0.001). Only old age and bulky disease were independent prognostic factors in HIV- negative cHL (p < 0.001, p = 0.046, respectively) (Table 4).

**Table 3:** Univariate survival analysis in HIV negative cHL patients

**Variable**	**cHL**			**MC subtype**		**NS subtype**	
	**N**	**OS**	**PFS**	**N**	**OS**	**N**	**OS**
**Demographic data**							
Age ≥50 yrs	97	<0.001	0.753	40	<0.001	57	0.156
Male sex	97	0.197	0.132	40	0.496	57	0.816
**Clinicopathologic data**							
IPS >2	94	0.025	0.046	38	0.029	56	0.964
Advanced stage	95	0.006	0.092	38	0.050	57	0.336
MC or NS subtype	97	0.012	0.144				
B symptom	91	0.377	0.735	37	0.812	54	0.365
Bulky disease	91	0.046	0.576	36	0.017	55	0.422
EBV infection	78	0.045	0.971	31	0.395	47	0.217
PB monocyte (≥7.1%)	90	0.652	0.402	35	0.813	55	0.076
**Immunohistochemical data**							
IDO (≥26%)	97	<0.001	0.828	40	0.017	57	0.017
CD163 (≥33%)	77	0.002	0.958	31	0.015	46	0.137
CD68 (≥5%)	73	0.058	0.314	29	0.078	44	0.536
CD4 (≥301 cells/mm^2^)	79	0.916	0.684	34	0.407	45	0.761
CD8 (≥2302 cells/mm^2^)	76	0.813	0.527	33	0.361	43	0.758
FOXP3/CD4 (≥0.5)	79	0.313	0.835	34	0.167	45	0.843

HIV, human immunodeficiency virus; cHL, classical Hodgkin lymphoma; N, number; OS, overall survival; PFS, progression free survival; IPS, international prognostic score; MC, mixed cellularity; NS, nodular sclerosis; PB, peripheral blood; IDO, indoleamine 2,3-dioxygenase.

**Table 4:** Multivariate survival analysis in classical Hodgkin lymphoma patients

**Variable**	**HIV- cHL**		**HIV- MC subtype**		**HIV- NS subtype**	
	**P**	**HR (95% CI)**	**P**	**HR (95% CI)**	**P**	**HR (95% CI)**
Age ≥50 years	<0.001	6.837 (2.588-18.062)	0.009	15.669 (1.995-123.042)	0.915	0.888 (0.099-7.967)
Advanced stage	0.403	1.584 (0.539-4.652)	0.329	2.263 (0.440-11.654)	0.141	3.382 (0.669-17.095)
Bulky disease	0.040	3.822 (1.060-13.783)	0.051	5.146 (0.991-26.725)	0.646	1.732 (0.166-18.091)
Blood monocyte	0.111	0.906 (0.802-1.023)	0.100	0.860 (0.719-1.029)	0.009	0.640 (0.459-0.894)
IDO	0.111	1.018 (0.996-1.040)	0.378	1.011 (0.98601.037)	0.001	1.104 (1.043-1.168)
CD163	0.812	1.002 (0.982-1.023)	0.439	1.008 (0.987-1.030)	0.020	1.061 (1.009-1.116)
CD68	0.636	0.970 (0.854-1.101)	0.906	0.992 (0.872-1.129)	0.735	1.056 (0.772-1.443)

HR, hazard ratio; CI, confidential interval; HIV, human immunodeficiency virus; cHL, classical Hodgkin lymphoma; HR, hazard ratio; CI, confidential interval; IDO, indoleamine 2,3-dioxygenase.

## Discussion

This is the first study to investigate the association of IDO expression with clinicopathologic characteristics of HL. We confirmed that IDO was positive in the background stromal cells of HL to a variable degree and that high expression of IDO was a significant prognostic predictor of unfavorable outcome in HL. Our results also provide a rationale for the development of novel therapeutic strategies targeting the tumor microenvironment, including immune cells, to achieve an optimal outcome in HL.

The understanding of the influence of cellular components on the clinical course of HL has progressively emerged over the last few years. Although the precise mechanisms required for these cells to orchestrate neoplastic and inflammatory reactions are not entirely understood, progress has been made by many researchers [12]. Recent studies have revealed that the tumor microenvironment (e.g., macrophage infiltration) is a major determinant of poor clinical outcome [12-16]. Our study results are in agreement with previous studies showing that macrophage rich HL correlates with poor outcome. Furthermore, we focused on the role of IDO secreted by stromal macrophages or dendritic cells on the clinicopathologic characteristics of HL.

In this retrospective review of 121 HL patients, we have shown that IDO expression was significantly higher in mixed cellularity subtype cases and HIV or EBV positive cases. Moreover, expression of IDO significantly correlated with old age, advanced Ann Arbor stage, and high IPS (Table 5). Overall, cases with high IDO expression corresponded to cases with relatively low immunity. IDO is involved in the production of immunomodulatory tryptophan metabolites that contribute to immunosuppression and immune tolerance. The cellular sources and the function of IDO are consistent with our findings.

**Table 5:** Characteristics of Hodgkin lymphoma patients according to IDO expression

**Variable**	**IDO high**	**IDO low**
Sex	Male	Female
Age group	Older	Young
Histologic type	MC, LD, LR	NLP, NS
Ann Arbor stages	Advanced stage	Limited stage
IPS	High	Low
B symptom	Present	Absent
EBV or HIV infection	positive	Not accompanied
FOXP3/CD4*	High	Low

*FoxP3/CD4 significantly correlated with IDO in cases with limited stages.

IDO, indoleamine 2,3-dioxygenase; MC, mixed cellularity; LD, lymphocyte-depleted; LR, lymphocyte-rich; NLP, nodular lymphocyte predominant; NS, nodular sclerosis; IPS, international prognostic score.

In our study, high IDO expression correlated with decreased CD4+ T cells and increased CD8+ T cells, and increased ratio of FoxP3/CD4 in HL of limited stages. This finding is partly consistent with previous reports, which suggest that tumors expressing high IDO had decreased numbers of tumor-infiltrating lymphocytes or increased numbers of FOXP3+ regulatory T cells (Treg) [7]. Also, Elpek et al. reported Treg dominated immune evasion in early stage B-cell lymphoma but not in late stage tumor [17]. Possibly, more complicated and various immune evasion mechanisms might exit in the microenvironment of advanced tumors. We hypothesize that the impairment of T cell immunity triggered by IDO contributes to the imbalance in the CD4/CD8 ratio and recruitment of Treg in HL. Further evaluation is needed to elucidate these mechanisms.

Currently, studies regarding the association between IDO and viral infection in tissue samples have rarely been attempted. In our study, IDO expression in EBV- or HIV-associated HL was considerably high. This finding merits some interpretation regarding the known characteristics of EBV or HIV infection in HL. Infection of EBV in East Asian HL patients occurs more frequently than in Western countries and is reported to be associated with a poor prognosis [18]. Activation of the transcription factor nuclear factor-kappa B (NF-kB) by EBV is known to be responsible for various immunologic changes in HL [19]. Paolo et al. suggested that non-canonical NF-kB activation is necessary for the induction of IDO expression [5]. Recently, Song et al. showed that EBV-induced IDO metabolites provide a potential mechanism by which EBV escapes immune attack by NK cells [20]. In addition, Manches et al. have reported that activation of NF-kB in HIV patients induces IDO expression in dendritic cells [21]. Therefore, correlation between EBV or HIV positivity and IDO expression in our results is explainable. It is noteworthy that all three HIV + HL in our series showed strikingly high IDO positivity. In HIV-negative cHL, EBV positivity was strongly associated with high expression of IDO, especially in MC subtypes. However, the overall expression level of IDO was very low regardless of EBV infection in NS subtype. Therefore, we suppose that IDO participates more significantly in the construction of microenvironment through recruitment of inflammatory cells rather than EBV does.

Over the past few years a considerable number of studies have been conducted on the prognostic effects of tumor infiltrating macrophages on the microenvironment of many solid tumors and hematolymphoid malignancies. We also observed that high infiltration of CD163+ macrophages and IDO were adverse prognostic factors of patient OS in univariate analysis. We separated groups of high IDO or histiocytes based on the cutoff values generated from ROC curves of automated analysis. However, the cutoff values we have chosen can be changed in other cohorts using conventional way of immunohistochemical assessment. Through the use of multivariate analysis, only IDO retained its statistical significance of negative prognostic indicator among total cohort (Additional file 2: Table S2), although the statistical power was weakened when confined in HIV-negative cHL. Therefore, we suspect that the prognostic impact of tumor associated macrophages on HL largely originates from the cells’ ability to secrete IDO. Our results are in agreement with many earlier studies which reported that IDO correlated with less favorable clinical outcomes.

The alleged protean immunomodulator IDO is now popularly recognized as an important factor in the tumor microenvironment for cancer immunity. Taken together, our findings provide evidence that IDO plays an important role in the microenvironment of HL.

## Conclusions

In summary, IDO was often highly expressed in the stromal cells of HL and correlated with poor prognostic factors, in addition to EBV or HIV infection. High expression of IDO was a significant negative predictor of patients’ survival. Our results provide evidence that IDO is a major immune modulator of HL.

## Competing interests

The authors declare that they have no competing interests.

## Authors’ contributions

JYC carried out acquisition of clinicopathologic data, analysis of immunohistochemical staining, and drafted the manuscript. JYY have made substantial contributions to acquisition of clinicopathologic data and interpreting it. YKJ have made substantial contributions to collect the data and analyze it. SHK have made substantial contributions to collect the data and analyze immunohistochemical staining. GSP have made substantial contributions to conception and design of this study. JRH have made substantial contributions to conception and design of this study. SHO carried out statistical analysis and revised the manuscript critically for important intellectual content. JEK have made the design and conception of this study and performed immunohistochemical staiing, interpreted the data, and revised the manuscript. All authors read and approved the final manuscript.

## Pre-publication history

The pre-publication history for this paper can be accessed here:

http://www.biomedcentral.com/1471-2407/14/335/prepub

## Supplementary Material

Additional file 1: Table S1 Association of EBV infection and clinicopathologic variables in HIV-negative classical Hodgkin lymphoma (HIV- cHL). Click here for file

Additional file 2: Table S2 Multivariate survival analyses in Hodgkin lymphoma patients. Click here for file
