# Supplementary material for: Indoleamine 2,3-dioxygenase (IDO) is frequently expressed in stromal cells of Hodgkin lymphoma and is associated with adverse clinical features: a retrospective cohort study
Source: BMC Cancer. 2014 May 15;14:335. doi: 10.1186/1471-2407-14-335 (PMC4026588; doi:10.1186/1471-2407-14-335)
Supplement: Additional file 1: Table S1 — Association of EBV infection and clinicopathologic variables in HIV-negative classical Hodgkin lymphoma (HIV- cHL). [file 1471-2407-14-335-S1.doc]

| **Additional file 1: Table S1. Association of EBV infection and clinicopathologic variables in HIV-negative classical Hodgkin lymphoma (HIV- cHL)** | | | | | | | | | |
| --- | --- | --- | --- | --- | --- | --- | --- | --- | --- |
| Variable (mean) | **HIV- cHL** | | | **HIV- cHL, MC** | | | **HIV- cHL, NS** | | |
|  | EBV+ | EBV- | P | EBV+ | EBV- | P | EBV+ | EBV- | P |
| Age (37.9years) | 42.5 | 35.5 | 0.067 | 53.5 | 49.0 | 0.522 | 29.7 | 30.5 | 0.867 |
| Male sex* | 28/37 | 26/48 | 0.045 | 16/20 | 9/13 | 0.681 | 12/17 | 17/35 | 0.152 |
| Advanced stage* | 21/37 | 21/48 | 0.278 | 14/20 | 10/13 | 1.000 | 7/17 | 11/35 | 0.544 |
| B symptom* | 15/35 | 9/42 | 0.052 | 10/20 | 2/10 | 0.235 | 5/15 | 7/32 | 0.481 |
| Bulky disease* | 2/33 | 5/42 | 0.456 | 1/18 | 2/11 | 0.539 | 7/17 | 11/35 | 0.544 |
| IPS (>2)* | 17/37 | 10/48 | 0.019 | 11/20 | 4/13 | 0.284 | 6/17 | 6/35 | 0.173 |
| IDO (10.3%) | 16.3 | 8.3 | 0.044 | 26.4 | 16.9 | 0.143 | 4.44 | 5.12 | 0.823 |
| CD163 (19.1%) | 24.7 | 17.0 | 0.142 | 32.3 | 29.4 | 0.803 | 16.6 | 12.3 | 0.341 |
| CD68 (3.3%) | 4.4 | 2.6 | 0.108 | 7.3 | 3.0 | 0.078 | 1.8 | 2.4 | 0.414 |
| FOXP3/CD4 (10.1%) | 8.0 | 6.8 | 0.827 | 37.2 | 33.0 | 0.902 | 1.6 | 5.1 | 0.454 |
| Values by Mann-whitney test (*; chi square test)  MC, mixed cellularity subtype; NS, nodular sclerosis subtype; P, p-value; EBV, Epstein-Barr virus; IPS, international prognostic score; IDO, indoleamine 2,3-dioxygenase;  The percentage of IDO, CD163, and CD68 was calculated by the area fraction of positive cells, whereas that of FOXP3 and CD4 was counted with the number of positive cells. | | | | | | | | | |
