# Supplementary material for: Indoleamine 2,3-dioxygenase (IDO) is frequently expressed in stromal cells of Hodgkin lymphoma and is associated with adverse clinical features: a retrospective cohort study
Source: BMC Cancer. 2014 May 15;14:335. doi: 10.1186/1471-2407-14-335 (PMC4026588; doi:10.1186/1471-2407-14-335)
Supplement: Additional file 2: Table S2 — Multivariate survival analyses in Hodgkin lymphoma patients. [file 1471-2407-14-335-S2.doc]

| **Additional file 2: Table S2. Multivariate survival analysis in Hodgkin lymphoma patients** | | | | | | | | |
| --- | --- | --- | --- | --- | --- | --- | --- | --- |
| Variable | **All cases** | | **HIV- cHL** | | **HIV- MC subtype** | | **HIV- NS subtype** | |
|  | P | HR (95% CI) | P | HR (95% CI) | P | HR (95% CI) | P | HR (95% CI) |
| Age ≥50 years | 0.020 | 3.102 (1.193-8.064) | <0.001 | 6.837 (2.588-18.062) | 0.009 | 15.669 (1.995-123.042) | 0.915 | 0.888 (0.099-7.967) |
| Advanced stage | 0.238 | 1.905 (0.653-5.558) | 0.403 | 1.584 (0.539-4.652) | 0.329 | 2.263 (0.440-11.654) | 0.141 | 3.382 (0.669-17.095) |
| Bulky disease | 0.150 | 2.601 (0.707-9.575) | 0.04 | 3.822 (1.060-13.783) | 0.051 | 5.146 (0.991-26.725) | 0.646 | 1.732 (0.166-18.091) |
| Blood monocyte | 0.224 | 0.931 (0.830-1.045) | 0.111 | 0.906 (0.802-1.023) | 0.100 | 0.860 (0.719-1.029) | 0.009 | 0.640 (0.459-0.894) |
| IDO | 0.011 | 1.023 (1.005-1.042) | 0.111 | 1.018 (0.996-1.040) | 0.378 | 1.011 (0.98601.037) | 0.001 | 1.104 (1.043-1.168) |
| CD163 | 0.796 | 1.003 (0.984-1.022) | 0.812 | 1.002 (0.982-1.023) | 0.439 | 1.008 (0.987-1.030) | 0.020 | 1.061 (1.009-1.116) |
| CD68 | 0.930 | 0.995 (0.889-1.114) | 0.636 | 0.970 (0.854-1.101) | 0.906 | 0.992 (0.872-1.129) | 0.735 | 1.056 (0.772-1.443) |
| HR, hazard ratio; CI, confidential interval; HIV- cHL, HIV negative classical Hodgkin lymphoma; MC, mixed cellularity; NS, nodular sclerosis; HR, hazard ratio; CI, confidential interval; IDO, indoleamine 2,3-dioxygenase | | | | | | | | |
